# Minimization of MEDA Biochip-Size in Droplet Routing

**DOI:** 10.3390/bios12050277

**Published:** 2022-04-27

**Authors:** Chiharu Shiro, Hiroki Nishikawa, Xiangbo Kong, Hiroyuki Tomiyama, Shigeru Yamashita

**Affiliations:** 1 Graduate School of Science and Engineering, Ritsumeikan University, Kusatsu 525-8577, Japan; chiharu.shiro@tomiyama-lab.org (C.S.); hiroki.nishikawa@tomiyama-lab.org (H.N.); kong@fc.ritsumei.ac.jp (X.K.); 2 Japan Society for the Promotion of Science Research Fellow, Tokyo 102-0083, Japan; 3 College of Information Science and Engineering, Ritsumeikan University, Kusatsu 525-8577, Japan; ger@cs.ritsumei.ac.jp

**Keywords:** digital microfluidics, MEDA, droplet routing, mathematical programming problem

## Abstract

With the increasing demand for fast, accurate, and reliable biological sensor systems, miniaturized systems have been aimed at droplet-based sensor systems and have been promising. A micro-electrode dot array (MEDA) biochip, which is one kind of the miniaturized systems for biochemical protocols such as dispensing, dilutions, mixing, and so on, has become widespread due to enabling dynamical control of the droplets in microfluidic manipulations. In MEDA biochips, the electrowetting-on-dielectric (EWOD) technique stands out since it can actuate droplets with nano/picoliter volumes. Microelectrode cells on MEDA actuate multiple droplets simultaneously to route locations for the purpose of the biochemical operations. Taking advantage of the feature, droplets are often routed in parallel to achieve high-throughput outcomes. Regarding parallel manipulation of multiple droplets, however, the droplets are known to be initially placed at a distant position to avoid undesirable mixing. The droplets thus result in traveling a long way for a manipulation, and the required biochip size for routing is also enlarged. This paper proposes a routing method for droplets to reduce the biochip size on a MEDA biochip with the allowance of splitting during routing operations. We mathematically derive the routing problem, and the experiments demonstrate that our proposal can significantly reduce the biochip size by 70.8% on average, compared to the state-of-the-art method.

## 1. Introduction and Related Work

### 1.1. Introduction

With the rapid widespread outbreak of the COVID-19 virus, an urgent requirement for fast, accurate, and reliable biochemical sensor systems has been created in recent years. The National Institute of Health (NIH) has launched the rapid acceleration of the diagnostic (RADx) initiative to develop and realize biochemical technologies for COVID-19 such as PCR testing [1]. One of the promising technologies has focused on miniaturized sensor systems aiming at nano/pico-scaled droplet-based microfluidics. In particular, the digital microfluidic biochip (DMFB) is attractive. Digital microfluidic biochip technology has been in the spotlight since it enables the automated manipulation of microliter fluid droplets for biochemical tests and measurements [2,3,4,5]. DMFBs have been extensively employed to accommodate biochemistry practices, such as point of care diagnostics, DNA sequencing, and so on [6,7,8,9]. Conventional DMFBs, however, have a drawback that they are not capable of controlling the volume and size of droplets during manipulation, and they encounter functionality issues for performing multiple manipulations at the same time.

To overcome such issues, an enhanced technology called micro-electrode dot array (MEDA) has emerged [10,11,12,13,14,15]. MEDA biochips, as well as DMFBs, use microelectrodes to manipulate droplets based on the electrowetting-on-dielectric (EWOD) principle. Unlike conventional DMFBs, a MEDA biochip allows microelectrode cells to transport multiple droplets with different sizes to dynamically dispense, mix, merge, stall, and split fluidic units, which provides high reconfigurability and programmability. In particular, routing of droplets is one of most significant manipulations since the droplets must be routed for any of the manipulations. On a MEDA biochip, inappropriate routing is liable to result in undesirable manipulations since the MEDA biochip does not have barriers to prevent the droplets from the interference. In order to avoid such undesirable manipulations, the droplets are kept apart from each other—at the initial placement and during routing. If a number of droplets need to be routed at the same time, the droplets must be carefully manipulated in order to not interfere with each other. In addition, the distance between the droplets needs to be long for increasing the size of the droplets. Therefore, the biochip size required for routing becomes large, resulting in prolonging its routing.

In this paper, we propose a routing method for homogeneous droplets, which allows for splitting a droplet into a couple of small droplets during routing. Our proposed routing method finds an optimal route in such a way that the biochip size necessary for routing is minimized. This paper is an extended version of the work in [16]. The contributions address that this work extends to utilized multiple couples of droplet routing while the previous work in [16] assumed the problem of minimizing the routing for mixing a couple of droplets. This extension enables us to achieve high throughput and aim at high reliability of biochemical procedures. The method proposed in this paper applies to practical examples of biochips [17,18,19], and contributes to the realization of biochips that ensure reliability and high throughput.

### 1.2. Related Work

In recent years, the usefulness of biochips has been greatly enhanced for biochemical protocol automation. One of the particular instances reported is a biochip developed to detect mercury ions (${Hg}^{2+}$), which is a major threat to the human body, in a simple but low-cost manner [17]. In addition, it is addressed that the collection of airborne bacteria and DNA extraction is realized by improvements of biochip technologies [3,18]. The research on ”organs-on-chips”, which reproduce the functions of human organs, is also in progress [19]. As mentioned above, there has been much work for practical applications of biochips. However, there is a challenging issue that biochips have been traditionally designed manually. For several years, design-level automation for biochips has been more attractive.

A DMFB is an example of a lab-on-a-chip that enables automation of biochemical experiments [20]. Over the past decade, DMFBs have been demonstrated for high-throughput biochemical experiments [21,22]. On a DMFB, nano/pico size liquids are manipulated as discrete droplets to have operations such as sensing, splitting, mixing, and dilution on a 2D array of microelectrodes. Cho et al. developed a droplet router for DMFB to achieve high performance [21]. This work attepmts to find a source-to-target route during transportation. Keszocze et al. proposed a routing problem considering temporary blockages, which are unavailable cells due to degradation, contamination, and so on [10]. They also developed a routing method to optimize the number of control pins [22]. Liang et al. attempted to find an otpimal route based on deep reinforcement learning techniques [23]. However, many DMFBs constrain the controllable droplet size as fixed, and real-time sensing of the state of droplets is difficult. Meanwhile, reliability also remains a challenging issue.

In order to deal with the issues above, MEDA biochips have been proposed [10,11,12,13,14,15,24,25,26,27,28]. The MEDA biochip consists of 0.048 mm${}^{2}$ of microelectrodes [29]. These microelectrodes are dynamically grouped, and the droplets are placed on them. The size of the droplet varies with the scale of the experiment. In general, the synthesis of MEDA biochips requires the processes: *Generate a sequential graph*, *Scheduling*, *Binding*, *Placement*, and *Routing* [24,25]. In particular, routing is the most critical process since it has a large impact on the overall synthesis results. The work in [11] is a routing time optimization method using an SAT (Boolean satisfiability testing) solver, which solves a routing problem with unavailable cells using the diagonal movement of droplets in MEDA. In case oof unavailable microelectrode cells, Liang et al. proposed a droplet routing framework based on multi-agent reinforcement learning [12]. The authors in [13] proposed a MEDA-based routing-based-synthesis (RBS) method. The RBS method actuates droplets to promote mixing, and the movement and mixing are conducted at the same time to reduce the manipulation time. In the RBS method, the droplets go around in a circle for efficient mixing. Due to the feature, the size of the biochip depends on the size of the droplet, and the number of cells is larger than that of other methods. The authors in [14] defined the problem of optimizing the source and target locations. It also solves routing on MEDA using node graphs and heuristics. The heuristic method has not been able to take full advantage of the MEDA biochip’s capabilities. The authors in [15] defined a source/target transport problem for multiple droplets in the presence of unavailable cells on a MEDA biochip and also proposed a method to eliminate the deadlock that occurs during multiple droplet transport. However, there was also a large difference in the transport completion time for each droplet to resolve the deadlock. This difference is caused by the poor positioning of the source and target. The methods of [14,15] needs two steps to solve the transport problem. First, source/target cells are determined by some factors. Second, the routing path is determined by avoiding deadlock for source/target cells. The work in [14,15] of fixing the source/target cell makes the problem inflexible. The use of a large number of cells means that errors due to faulty operation in each cell are also likely to occur [26]. The method of [26,27] involves moving the droplet after it has been broken down to its maximum particle size. This method works best when there are many unavailable cells. On the other hand, if there are less than a certain number of unavailable cells, the routing time may increase compared to normal droplet transport due to the time required for separation and recombination.

Howladar proposed a homogeneous droplet routing method for the shortest path problem [15]. The shortest path problem is to find a route that minimizes the number of cells used on the routes that a droplet can travel in a certain time. The drawbacks of [14,15] are that routing with multiple droplets at the same time requires a number of dispensing units. The dispensing units are relatively larger than a microelectrode cell and are placed at a distant from each other to avoid the interference, resulting in prolonging the transportation time and enlarging the biochip size. In addition, Roy et al., in [28], proposed a routing with split, mix, and reshape to exploit the potential of MEDA biochips and addressed fine-grained droplet manipulations; taking advantage of the MEDA biochips is crucial to achieving high-performance and reliability. These factors encourage the motivation of a routing method that allows for splitting into multiple droplets during routing to deal with the shortcomings of the MEDA biochips [16,30].

The rest of this paper is organized as follows. Section 2 proposes a droplet routing method considering split manipulation during routing. Section 3 describes the experiments and comparison, and Section 4 concludes this paper.

## 2. Droplet Routing for Minimization of MEDA Biochip-Size

### 2.1. Problem Definition and Example

We define the droplet routing problem in MEDA biochips as follows: our routing problem in this paper assumes parallel homogeneous droplet routing with splitting operation. The problem assumes a couple of droplets be transported for mixing, and we allow the droplets to be split during routing. Given the number and size of droplets with a maximum limit of biochip size as a constraint, our routing determines the best routes such that the biochip size, which represents the rectangle constructed with the routing paths by the droplets, is minimized.

**Figure 1:**
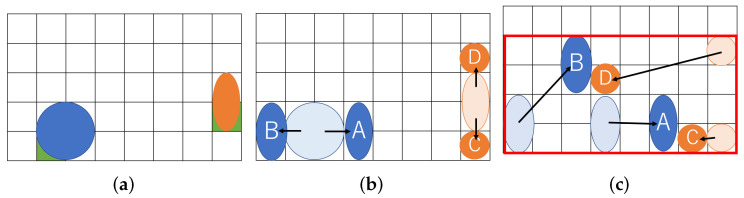
Routing example for a proposed method. (**a**) Initial position of droplets; (**b**) Split into small droplets; (**c**) Droplet position after routing.

Figure 1 shows an example of our routing problem aware of dilution. Figure 1a represents a couple of droplets to be mixed on a MEDA biochip. The (8 × 5) rectangle indicates the biochip cells, and the two circles in blue and orange represent droplets. The blue droplet is called buffer and the orange one is called sample. The droplets initially occupy four and two cells, respectively. In order to address the coordinate of the cell, we define the reference point, which is green in the lower left corner of the droplet [27]. In this paper, we assume that each droplet is allowed to be split into two small droplets during routing. Figure 1b shows the split operation. If a droplet is an input without splitting, it must be input at a position far from the one shown in Figure 1b due to reservoir interference [31]. The sample droplet is split into *A* and *B* small droplets, and the buffer droplet is split into *C* and *D*. Assume that the size of each droplet is (A,B,C,D)=(2,2,1,1) and consider routing droplets to mix *A* and *C* and to do *B* and *D* as shown in Figure 1c. We show the routing path of the droplets and create a bounding box of the paths in Figure 1c. As shown in Figure 1c, the (8 × 4) rectangle on the biochip are required for transporting the droplets.

**Figure 2:**
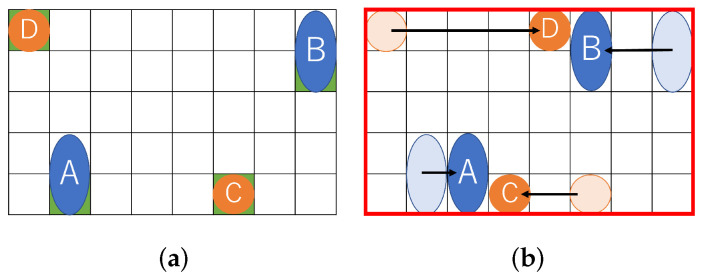
Routing example for a state-of-the-art method. (**a**) Initial position of droplets; (**b**) Droplet position after routing.

Figure 2 shows a similar problem solved with existing a state-of-the-art method. A certain distance must be kept between droplets due to the dispensing unit. Figure 2a shows the initial position of the droplets assuming the constraints of the dispensing unit. After that, the droplets move for mixing. Figure 2b shows the positional relationship of each droplet after the droplet is moved. As shown in Figure 2b, the (8 × 5) rectangle on the biochip is required for transporting the droplets.

In the example, we had several assumptions towards the droplets. As shown in Figure 1b, the split droplets must be distant by a boundary width, and not interfere with each other. The droplets must keep a distance during transportation as well, otherwise the droplets obtain undesirable mixing. For simplicity, we neglect the density and volume errors of the droplets due to the contamination on the routing path by the droplets. Since the problem aims at the minimization of biochip size (the total size of the rectangle constructed by droplets passing), we do not take into account the speed of droplets.

### 2.2. Formulation

We formulate the problem for parallel droplet routing with splitting. Table 1 shows the notations used in the following formulation.

Formula (1) represents the constraint on the coordinates when the droplets are dispensed into the MEDA biochip [27]. $\left({x\_init}_{i,0},{y\_init}_{i,0}\right)$ is the reference point (x,y) at the time of input of droplet *i*. Let *t* denote the time, and the droplet is input to the MEDA biochip at t=0. $\left({w\_init}_{i},{h\_init}_{i}\right)$ is assumed to be the aspect ratio of droplet *i*, and we assume the shape of the droplets cannot be changed. At t=0, the droplets must be placed on an edge of the biochip.

(1) $$\begin{array}{ccc} \forall i,\; & & \left({x\_init}_{i,0}=1\right)\;\vee \;\left({x\_init}_{i,0}+{w\_init}_{i}=W+1\right)\\ & & \;\vee \;\left({y\_init}_{i,0}=1\right)\;\vee \;\left({y\_init}_{i,0}+{h\_init}_{i}=H+1\right) \end{array}$$

Formula (2) represents the volume and shape of the droplet [15]. The aspect ratio of the droplet at the input is constant until it is split after it is determined by Formula (2). This constraint guarantees that the aspect ratio of a droplet is kept unchanged over time. ${w\_init}_{i},{h\_init}_{i}$ is the vertical and horizontal size of droplet *i* before splitting.

(2) $$\forall i,\;{w\_init}_{i}\times {h\_init}_{i}={Input\_Vol}_{i}$$

**Figure 3:**
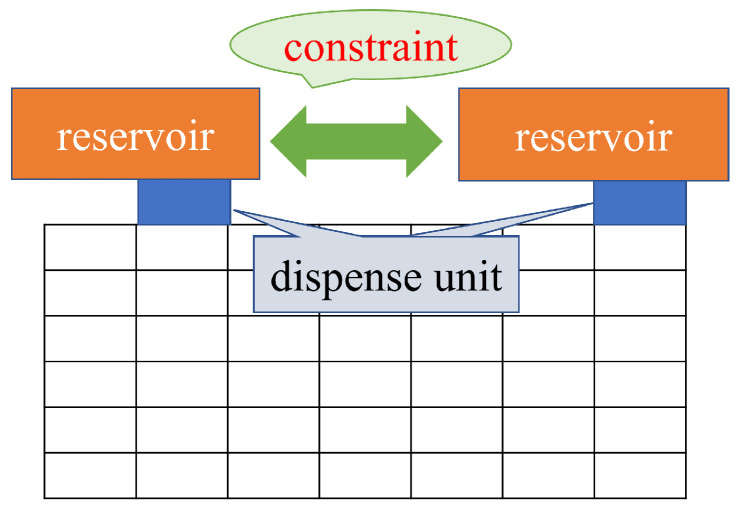
Distance between reservoirs.

Formula (3) shows the constraints on the dispensing units for input to avoid fluidic leakage and dispensing clustering. Formula (3) determines the initial position of droplets. Considering the size of the dispensing units and the interference of the reservoir, the distance between the dispensing units must be greater than a certain value $L_{RR}$ [27,31]. Determining the initial position of the droplet is important because the biochip size is affected by the constraints of the dispensing unit. In this paper, the distance between two dispensing units is defined as the sum of twice the maximum edge of the droplet and the distance *B*. The formula (3) constrains its boundary width, as shown in Figure 3.

$$\begin{array}{c} \forall i1,i2,j,(i1\neq i2),\; \end{array}$$

$$\left(\begin{array}{cc} \;|{x\_init}_{i1,0}-{x\_init}_{i2,0}\left|+\right|{y\_init}_{i1,0}-{y\_init}_{i2,0}|\\ \geq \;{max}_{i}\left(2\times {w\_init}_{i}+B\right) & \; \end{array}\right)$$

(3) $$\wedge \left(\begin{array}{cc} \;|{x\_init}_{i1,0}-{x\_init}_{i2,0}+\left|{y\_init}_{i1,0}-{y\_init}_{i2,0}\right|\\ \geq \;{max}_{i}\left(2\times {h\_init}_{i}+B\right) & \; \end{array}\right)$$

**Figure 4:**
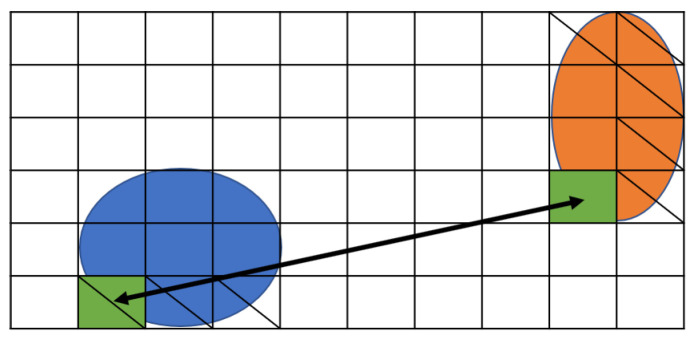
Boundary width between the droplets.

Figure 4 shows the position of the droplet that satisfies Formulas (1) and (3). The shaded area in Figure 4 represents Formula (1).

The reference point when the droplet is transported before splitting is represented by Formula (4) [15]. ${pre\_move}_{i,t}$ is a 0–1 variable.

If droplet *i* moves before split at time *t*, ${pre\_move}_{i,t}$ becomes 1. Droplet *i* does not move before the split at time *t*, otherwise ${pre\_move}_{i,t}$ becomes 0. Unlike DMFBs, Droplets on the MEDA biochip can move not only in the horizontal and vertical directions, but also in the diagonal direction.

$$\begin{array}{c} \forall i,t,\;if\;({pre\_move}_{i,t}=1)\;\; \end{array}$$

$$\begin{array}{c} \left\{\left({x\_init}_{i,t+1}={x\_init}_{i,t}\right)\vee \left({x\_init}_{i,t+1}={x\_init}_{i,t}+1\right)\vee \left({x\_init}_{i,t+1}={x\_init}_{i,t}-1\right)\right\} \end{array}$$

(4) $$\begin{array}{c} \wedge \;\{\left({y\_init}_{i,t+1}={y\_init}_{i,t}\right)\vee \left({y\_init}_{i,t+1}={y\_init}_{i,t}+1\right)\vee \left({y\_init}_{i,t+1}={y\_init}_{i,t}-1\right)\} \end{array}$$

Formula (5) shows how to determine the aspect of the droplet after splitting. The aspect ratio of the droplet after splitting on the MEDA biochip remains fixed. Formula (5) expresses the relationship between droplet volume and droplet shape. This constraint also guarantees that the aspect ratio of a droplet is kept unchanged over time.

(5) $$\forall i,j,\;\left(0\leq t\right)\;{w\_mix}_{i,j}\times {h\_mix}_{i,j}={Vol}_{i,j}$$

When the droplets are split, Formula (6) represents the constraint for the case where the two droplets move up and down after a split, and Formula (7) represents the constraint for the case where the two droplets move left and right after the split. ${pre}_{i}$ represents the time when the split of droplet *i* starts. ${w\_mix}_{i,j,t}$ and ${h\_mix}_{i,j,t}$ are the horizontal and vertical sizes of droplets i,j at time *t*. ${x\_aliquot}_{i,j}$ and ${y\_aliquot}_{i,j}$ are the coordinates of the reference point for the two of small droplets *i* and *j* after splitting.

$$\begin{array}{c} \forall i,j,{pre}_{i}\left\{{pre}_{i}={max}_{t}\left(t\times {pre\_move}_{i,t}\right)\right\}\;\\ if\left({w\_init}_{i}={w\_mix}_{i,j}\right)\; \end{array}$$

(6) $$\sum_{j1,j2(j1\neq j2)}^{Num\_droplet}\left\{\begin{array}{c} \;\left({y\_aliquot}_{i,j2}={y\_init}_{i,{pre}_{i}}+{h\_mix}_{i,j1}+B\right)\\ \;\wedge \;({y\_aliquot}_{i,j1}={y\_init}_{i,{pre}_{i}}-B) \end{array}\right\}$$

$$\begin{array}{c} \forall i,j,{pre}_{i}\left\{{pre}_{i}={max}_{t}\left(t\times {pre\_move}_{i,t}\right)\right\}\;\\ if\left({h\_init}_{i}={h\_mix}_{i,j}\right)\; \end{array}$$

(7) $$\sum_{j1,j2(j1\neq j2)}^{Num\_droplet}\left\{\begin{array}{c} \;\left({x\_aliquot}_{i,j2}={x\_init}_{i,{pre}_{i}}+{w\_mix}_{i,j1}+B\right)\\ \;\wedge \;({x\_aliquot}_{i,j1}={x\_init}_{i,{pre}_{i}}-B)\} \end{array}\right\}$$

**Figure 5:**
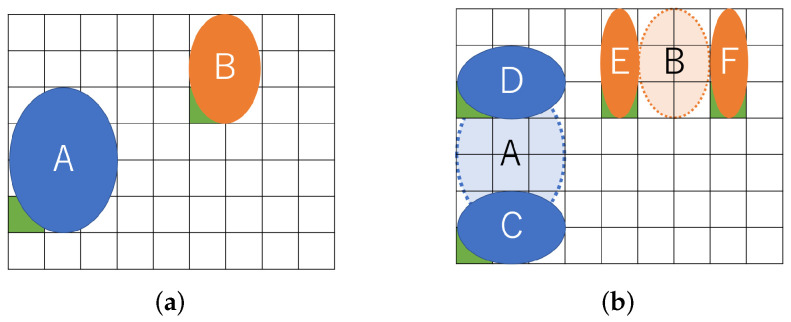
Location until routing starts. (**a**) Input droplets; (**b**) Splitting droplets.

Figure 5 shows the movement of droplets according to Formulas (6) and (7). There are two droplets, as shown in Figure 5a, and sample droplet *A* is vertically split into two small droplets *C* and *D* in Figure 5b. Droplet *B* is horizontally split to form droplet *E* and *F*. At splitting, the small droplets have to be distant more than the boundary width to avoid interference.

In Formula (8), let ${x\_mix}_{i,j,t}$ and ${y\_mix}_{i,j,t}$ denote the coordinates of small droplets at time *t*. ${x\_mix}_{i,j,t}$ and ${y\_mix}_{i,j,t}$ represent the horizontal and vertical coordinates of the *j*-th droplet by the original droplet *i*, respectively. According to [32,33,34], aliquot operation is assumed to take a second for splitting. Therefore, the time during aliquot operation is fixed as a certain time Aliquot, and which is taken into account in Formula (8).

(8) $$\begin{array}{c} \forall i,j,t,\{1\leq t\leq Aliquot+{max}_{t}\left(t\times {pre\_move}_{i,t}\right)\}\;\\ \left({x\_mix}_{i,j,t}={x\_aliquot}_{i,j}\right)\wedge \left({y\_mix}_{i,j,t}={y\_aliquot}_{i,j}\right) \end{array}$$

**Figure 6:**
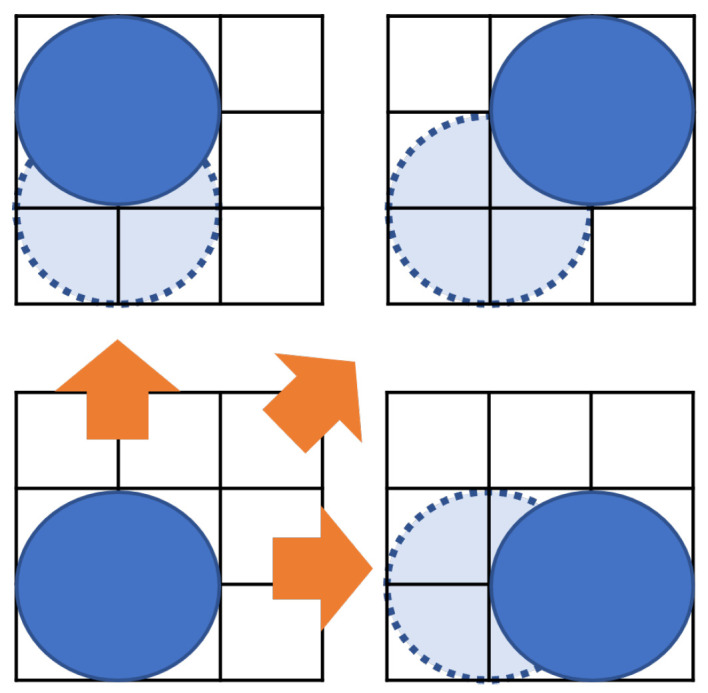
Position of droplets at input.

As shown in Figure 6, recall that the droplets on the MEDA biochip can move in the vertical, horizontal, and diagonal directions [25,27]. Formula (9) represents the constraint that the droplet (i,j) moves.

$$\begin{array}{c} \forall i,j,k,t,\;(t\geq {time\_move}_{i,j})\; \end{array}$$

$$\begin{array}{c} \left\{\left({x\_mix}_{i,t+1}={x\_mix}_{i,t}\right)\;\vee \left({x\_mix}_{i,t+1}={x\_mix}_{i,t}+1\right)\;\vee \left({x\_mix}_{i,t+1}={x\_mix}_{i,t}-1\right)\right\} \end{array}$$

(9) $$\begin{array}{c} \wedge \;\{\left({y\_mix}_{i,t+1}={y\_mix}_{i,t}\right)\;\vee \left({y\_mix}_{i,t+1}={y\_mix}_{i,t}+1\right)\;\vee \left({y\_mix}_{i,t+1}={y\_mix}_{i,t}-1\right)\} \end{array}$$

Formula (10) represents static and dynamic constraints to prevent undesirable mixing and interference between droplets [15]. Let $d_{1}$ and $d_{2}$ denote 0–1 decision variables. When $d_{1}=d_{2}$, Formula (10) represents static constraints, and when $d_{1}\neq d_{2}$, Formula (10) represents dynamic constraints.

$$\begin{array}{c} \forall i1,i2,j1,j2,t,d_{1},d_{2},\;\left(\;d_{1},d_{2},=0\;or\;1\;\right)\;\;\\ \;if(j1\neq j2) \end{array}$$

(10) $$\begin{array}{cc} \; & \left\{{y\_mix}_{i1,j1,t-d_{1}}-\;\left({y\_mix}_{i2,j2,t-d_{2}}+{h\_mix}_{i2,j2}\right)\geq B+1\right\}\\ \; & \;\wedge \;\{{x\_mix}_{i1,j1,t-d_{1}}-\;\left({x\_mix}_{i2,j2,t-d_{2}}+{w\_mix}_{i2,j2}\right)\geq B+1\} \end{array}$$

In this problem, we are routing the two couples of droplets until they both start mixing. Formula (11) calculates the biochip size required for the routing operation up to this point.

(11) $$\begin{array}{cc} S\;= & \{{max}_{i,j,k}\left({x\_mix}_{i,j,k}+{w\_mix}_{i,j}\right)-{min}_{i,j,k}\left({x\_mix}_{i,j,k}\right)\}\\ \; & \times \{{max}_{i,j,k}\left({y\_mix}_{i,j,k}+{h\_mix}_{i,j}\right)-{min}_{i,j,k}\left({y\_mix}_{i,j,k}\right)\} \end{array}$$

These are the formulations that we have realized in this paper. When the aspect ratio of the droplet is freely varied in [10], Formulae (2) and (5) are changed as in Formulas (12) and (13). To avoid unavailable cells, a change in the aspect ratio of the droplet is used. In this method, there are no unavailable cells, so it is not implemented.

(12) $$\forall i,t,\;{w\_init}_{i,t}\times {h\_init}_{i,t}={W\_pre\_init}_{i}\times {H\_pre\_init}_{i}$$

(13) $$\forall i,j,t,\left(0\leq t\right)\;{w\_mix}_{i,j,t}\times {h\_mix}_{i,j,t}={Vol}_{i,j}\;$$

### 2.3. Multiple Couples of Droplets Routing

Homogeneous droplet routing generally asks for the accurate density and volume to achieve high reliability in manipulations such as dilution and mixing. In the real world, however, the microelectrode degradation and contamination may lead to mixing failure. In many cases, error recovery systems try to separate the mixed droplet into independent original droplets, modify the density and volume, and attempt to accurately remix them with a goal of improving the reliability [35].

Another approach to high reliability in mixing is to conduct multiple mixing operations in parallel. For example, consider the mixing operation for droplet (*A* + *C*) and droplet (*B* + *D*) in Figure 1c of the previous subsection again. Even if mixing of (*A* + *C*) is failed due to the microelectrode degradation or contamination on the cells, the other mixing (*B* + *D*) may be conducted accurately. If both mixing operations fail, the operations are iteratively conducted until mixing has been accomplished. However, such a scenario unfortunately results in low throughput outcomes. This section extends the proposed method in the previous section to use a number of couples of droplets for high reliability.

**Figure 7:**
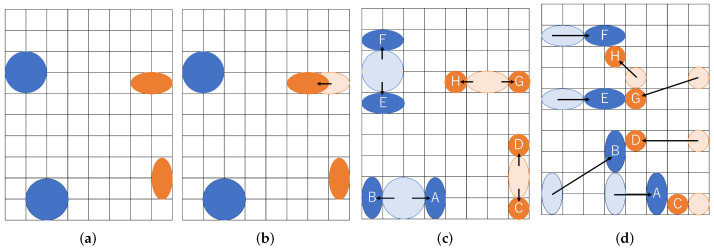
Multiple couples of droplets routing. (**a**) Input droplets; (**b**) Transportation before splitting; (**c**) Splitting droplets; (**d**) Transportation of small droplets.

We give an example of our multiple couples of droplets routing in Figure 7. Two couples of droplets are transported to be mixed. Each mixing operation is aimed at two cells of the buffer droplet and four cells of sample droplet. As illustrated in the figure, each size of the droplets is represented as (A,B,C,D,E,F,G,H)=(2,2,1,1,2,2,1,1), and droplets are routed to mix *A* and *C*, *B* and *D*, *E* and *G*, and *F* and *H*, respectively. The resultant route in the example is shown in Figure 7d. The split operation transitions from the state in Figure 7a to the state in Figure 7c through the state in Figure 7b. Figure 7a shows the positional relationship immediately after input from the dispensing unit. In Figure 7c, the droplets are separated by the boundary width, which is the distance where they do not interfere with each other.

Multiple couples of droplet routing are mainly based on the proposed method addressed in the previous section.

We can easily realize the routing method with the transformation from Formula (1) into Formula (14). The subscript *s* indicates an integer for the total kinds of droplets.

(14) $$\begin{array}{ccc} \forall s,i,\; & & \left({x\_init}_{s,i,0}=1\right)\;\vee \;\left({x\_init}_{s,i,0}+{w\_init}_{s,i}=W+1\right)\\ & & \;\vee \;\left({y\_init}_{s,i,0}=1\right)\;\vee \;\left({y\_init}_{s,i,0}+{h\_init}_{s,i}=H+1\right) \end{array}$$

## 3. Experiments

### 3.1. Setup

We have conducted the experiments to demonstrate the effectiveness of our proposed method in simulation. We give the kind, number, and size of droplets, and the couples of droplets to be mixed are determined before routing. We assume that all droplets move at the same speed. Droplet velocity does not affect biochip size. The initial position of the droplet is determined according to the constraints of the dispensing unit. The goal of the routing problem is to minimize the biochip size on the biochip. We compare the biochip size with the following two methods.

- A state-of-the-art method presented in [15], which initially inputs four/eight droplets onto the biochip for each experimental scenario.
- The proposed method inputs two/four droplets and splits them into four/eight droplets for each experimental scenario.

In the experiments, we are given the size of the biochip as (200×200). The experiments have been conducted on Intel Core i9-7980XE 2.60GHz with 128GB memory. IBM ILOG CPLEX Optimization Studio 20.1.0 is used for both the state-of-the-art method and the proposed method.

In the experiments, we assume two experimental scenarios. The former experimental scenario is a couple of droplets routing to create two resultant droplets, and the second experiment is multiple couples of droplets routing to create four resultant droplets by mixing operations. In this experimental scenario, the size of each droplet is assumed to be given randomly, which is shown in Table 2. This scenario assumes to solve the problem for mixing of droplets *A* and *C* and of droplets *B* and *D*. The runtime is limited by up to 10 h in CPU time. If an optimal solution is failed to be found within the runtime, the solution at the time limit is employed for comparison.

Similarly, routing of multiple couples of droplets assumes that the size of each droplet is given, as shown in Table 3. Unlike routing of a couple of droplets, this scenario assumes multiple couples of droplets go into mixing. The limit of the runtime is set to 50 h due to considering the computational complexity of the problem.

### 3.2. Results

**Figure 8:**
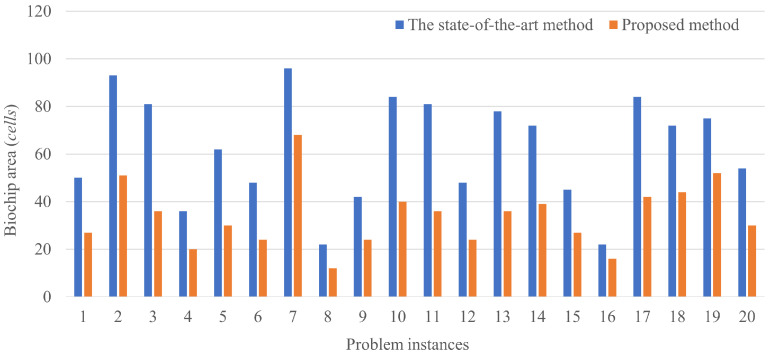
Results of a couple of droplet routings.

The results of routing a couple of droplets are shown in Figure 8. The horizontal axis shows the problem instances and the vertical axis shows the biochip size, where the total number of the cells is passed by the droplets.

**Figure 9:**
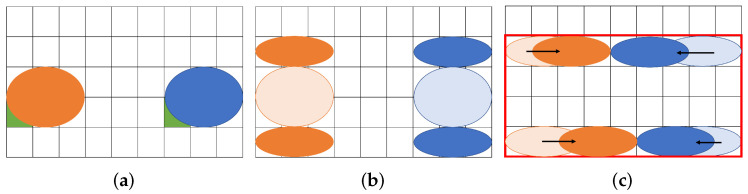
Trajectory of droplet movement in the proposed method. (**a**) Input droplets; (**b**) Split droplets; (**c**) Finish routing.

**Figure 10:**
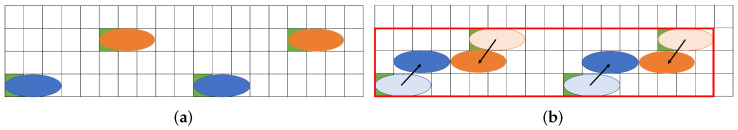
Trajectory of droplet movement in the state-of-the art method. (**a**) Input droplets; (**b**) Finish routing.

Figure 9 and Figure 10 show the trajectory of droplet movements in Case 1. Figure 9 shows the trajectory of Case 1 solved using the proposed method. The biochip size can be calculated to be 36(9×4). Figure 10 shows the trajectory of Case 1 solved by the state-of-the art method. The biochip size can be calculated to be 51(17×3). All the results show that the biochip size of the proposed method achieved less than the state-of-the-art method. The proposed method approximately reduces the biochip size by 56.3% on average. The results are attributed to the distance between dispensing units of the droplets in the state-of-the-art method. If the multiple droplets are independently input by dispensing units, the input droplets are distant due to the placement constraint of the dispensing units. Otherwise, the input droplets are interfered by each other, resulting undesirable manipulations as the error. On the other hand, if large droplets are initially input, the boundary width between the droplets is necessary to be distant to avoid the interference. However, even taking into account the effect of such distances, splitting operation of the droplets during routing by our proposed method can achieve the reduction of the biochip size, compared to the state-of-the-art method. The constraint on the initial position of the droplet is related to the biochip size. The closer the initial position of the droplets are to the mixing droplets, the smaller the biochip size is.

**Figure 11:**
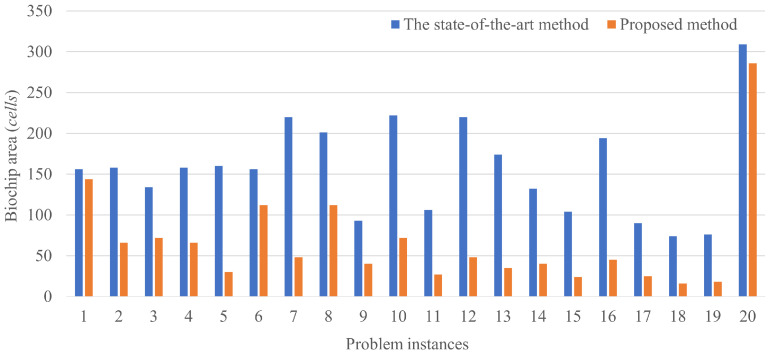
Results of multiple couples of droplets routing.

Figure 11 shows the results of multiple couples of droplet routing. Even if the number of droplets is increased, our proposed method demonstrates the effectiveness compared to the state-of-the-art method. The results show that the biochip size obtained by the proposed method is reduced by up to 81.4% and by 56.2% on average. Specifically, Case 1 and Case 20 show that our method achieves the slight reduction of the biochip size. As aforementioned, the state-of-the-art method fails to reduce the biochip size in the case with the large droplets.

Through these experiments, we demonstrate that the proposed method outperforms the state-of-the-art method. With the reduction of the biochip size, we imply that other manipulations will be conducted. In addition, the microelectrode degradation and contamination on the cells are taken into account in the future.

## 4. Conclusions

We have proposed the routing method to reduce the biochip size, allowing the splitting operation during routing. In addition, we extend our proposed method to multiple couples of droplet routing to aim at high reliability and throughput by exploiting the potential of the MEDA biochip. In the experimental results, our proposed method outperforms the state-of-the-art method and dramatically reduces the biochip size in routing.

We imply that our contribution enables other biochemical manipulations at the same time on the biochip. The proposed method, however, additionally requires splitting operations unlike the state-of-the-art method, and the operation may produce the error of the density and volume. In the future, we plan to deal with the issues above. Moreover, we will develop error-tolerant techniques for droplet routing.

## Figures and Tables

**Table 1:** Input data/Notations.

Number of input droplets	Num_droplet
Chip size	W×H
Input droplet size	${Input\_Vol}_{i}$
Target volume after split	${Vol}_{i,j}$
Distance to avoid interference	*B*
Time required for the split operation	Aliquot

**Table 2:** The volume of droplets for droplet routing problems.

Case	The State-of-the-Art Method [15]				Proposed Method	
	Droplet *A*	Droplet *B*	Droplet *C*	Droplet *D*	Droplet (*A* + *B*)	Droplet (*C* + *D*)
1	3	3	3	3	6	6
2	6	6	8	8	12	16
3	2	2	8	8	4	16
4	2	2	2	2	4	4
5	3	3	4	4	6	8
6	2	2	3	3	4	6
7	8	8	8	8	16	16
8	1	1	1	1	2	2
9	1	1	4	4	2	8
10	4	4	8	8	8	16
11	8	8	2	2	16	4
12	3	3	2	2	6	4
13	8	8	1	1	16	2
14	6	6	4	4	12	8
15	4	4	2	2	8	4
16	1	1	2	2	2	4
17	3	3	8	8	6	16
18	4	4	6	6	8	12
19	6	6	6	6	12	12
20	4	4	4	4	8	8

**Table 3:** The volume of droplets for multiple couples of droplet routing problems.

	Droplet	The State-of-the-Art Method [15]								Proposed Method			
Case		*A*	*B*	*C*	*D*	*E*	*F*	*G*	*H*	(*A* + *B*)	(*C* + *D*)	(*E* + *F*)	(*G* + *H*)
1		4	4	6	6	4	4	6	6	8	12	8	12
2		1	1	5	5	1	1	5	5	2	10	2	10
3		4	4	3	3	4	4	3	3	8	6	8	6
4		1	1	5	5	1	1	5	5	2	10	2	10
5		5	5	2	2	5	5	2	2	10	4	10	4
6		6	6	3	3	6	6	3	3	12	6	12	6
7		7	7	4	4	7	7	4	4	14	8	14	8
8		6	6	8	8	6	6	8	8	12	16	12	16
9		4	4	2	2	4	4	2	2	8	4	8	4
10		5	5	7	7	5	5	7	7	10	14	10	14
11		3	3	3	3	3	3	3	3	6	6	6	6
12		7	7	4	4	7	7	4	4	14	8	14	8
13		8	8	1	1	8	8	1	1	16	2	16	2
14		1	1	6	6	1	1	6	6	2	12	2	12
15		3	3	2	2	3	3	2	2	6	4	6	4
16		5	5	6	6	5	5	6	6	10	12	10	12
17		1	1	4	4	1	1	4	4	2	8	2	8
18		1	1	2	2	1	1	2	2	2	4	2	4
19		2	2	2	2	2	2	2	2	4	4	4	4
20		7	7	8	8	7	7	8	8	14	16	14	16

## Data Availability

Not applicable.
